# Research priorities in children and adults with congenital heart disease: a James Lind Alliance Priority Setting Partnership

**DOI:** 10.1136/openhrt-2022-002147

**Published:** 2022-11-22

**Authors:** Nigel E Drury, Clare P Herd, Giovanni Biglino, Katherine L Brown, Louise Coats, Michael J Cumper, Rafael R Guerrero, Alex Miskin, Sarah Murray, Fraser Pender, Sasha Rooprai, John M Simpson, John D R Thomson, Jara Weinkauf, Julie Wootton, Timothy J Jones, Katherine Cowan

**Affiliations:** 1 Institute of Cardiovascular Sciences, University of Birmingham, Birmingham, UK; 2 Paediatric Cardiac Surgery, Birmingham Children's Hospital, Birmingham, UK; 3 Institute of Applied Health Research, University of Birmingham, Birmingham, UK; 4 Bristol Medical School, University of Bristol, Bristol, UK; 5 National Heart and Lung Institute, Imperial College London, London, UK; 6 Cardiac Intensive Care Unit, Great Ormond Street Hospital for Children, London, UK; 7 Institute of Cardiovascular Science, University College London, London, UK; 8 Adult Congenital Heart Unit, Freeman Hospital, Newcastle upon Tyne, UK; 9 Population Health Sciences Institute, Newcastle University, Newcastle upon Tyne, UK; 10 Patient or Parent, UK; 11 Department of Paediatric Cardiac Surgery, Alder Hey Children's Hospital, Liverpool, UK; 12 Faculty of Health and Life Sciences, University of Liverpool, Liverpool, UK; 13 Department of Paediatric Cardiology, Evelina London Children's Hospital, London, UK; 14 School of Biomedical Engineering & Imaging Sciences, King's College London, London, UK; 15 Department of Paediatric Cardiology, Leeds General Infirmary, Leeds, UK; 16 Department of Paediatric Cardiology, Johns Hopkins Children's Center, Baltimore, Maryland, USA; 17 James Lind Alliance, Southampton, UK

**Keywords:** heart defects, congenital, research design, delivery of health care

## Abstract

**Objective:**

To bring together patients, parents, charities and clinicians in a Priority Setting Partnership to establish national clinical priorities for research in children and adults with congenital heart disease.

**Methods:**

The established James Lind Alliance methodology was used to identify and prioritise research on the management of congenital heart disease, focusing on diagnosis, treatment and outcomes. An initial open survey was used to gather potential uncertainties which were filtered, categorised, converted into summary questions and checked against current evidence. In a second survey, respondents identified the unanswered questions most important to them. At two final workshops, patients, parents, charities and healthcare professionals agreed the top 10 lists of priorities for child/antenatal and adult congenital heart disease research.

**Results:**

524 respondents submitted 1373 individual questions, from which 313 out of scope or duplicate questions were removed. The remaining 1060 questions were distilled into summary questions and checked against existing literature, with only three questions deemed entirely answered and removed. 250 respondents completed the child/antenatal survey (56 uncertainties) and 252 completed the adult survey (47 uncertainties). The questions ranked the highest by clinicians and non-clinicians were taken forward to consensus workshops, where two sets of top 10 research priorities were agreed.

**Conclusions:**

Through an established and equitable process, we determined national clinical priorities for congenital heart disease research. These will be taken forward by specific working groups, a national patient and public involvement group, and through the establishment of a UK and Ireland network for collaborative, multicentre clinical trials in congenital heart disease.

**WHAT IS ALREADY KNOWN ON THIS TOPIC**

- There is a lack of evidence to inform clinical decision-making in children and adults with congenital heart disease. Priority Setting Partnerships provide an equitable mechanism for identifying and prioritising research that is important to patients, their families and clinicians, through shared decision-making.

**WHAT THIS STUDY ADDS**

- Through an established, multistakeholder, consensus development process, this partnership determined national priorities for research in children and adults with congenital heart disease, providing a platform for conducting the research that matters most to those who may directly benefit.

**HOW THIS STUDY MIGHT AFFECT RESEARCH, PRACTICE OR POLICY**

- This work will provide a catalyst for collaborative congenital heart disease research in the United Kingdom. A national strategy has been developed to address the priorities, including the establishment of a UK and Ireland network for conducting multicentre studies, especially clinical trials, a national patient and public involvement group, and specific working groups focusing on one or more related priorities.

## Introduction

Congenital heart disease (CHD) is the most common type of birth defect, affecting 13 children born every day in the UK,^1^ but only around half are detected on routine antenatal screening.^2^ Many children require open heart surgery or other intervention, often within the first year of life and survival continues to improve^2 3^; yet early morbidities remain common after surgery^4^ and infants have an ongoing risk of death following discharge.^5^ Nowadays around 97% of children born with CHD are expected to survive into adulthood^6^ and there are estimated to be over 250 000 adults living with CHD in the UK,^7^ including an emerging population with conditions that previously were rarely compatible with survival, presenting new diagnostic and therapeutic challenges. Almost all patients require lifelong follow-up in specialist clinics to manage their unresolved burden of disease^8^; many develop late complications related to their condition or previous procedures, and have an ongoing need for increasingly hazardous reinterventions, to treat recurrent or progressive lesions, or replace prosthetic implants such as valves, conduits or pacemakers. Uncertainty over further interventions and long-term outlook may contribute to increased levels of anxiety, depression and other mental health issues in CHD.^9^ While a growing number are living with CHD into their 60s and beyond, developing acquired morbidities and senescence,^10^ long-term survival remains reduced compared with the general population, especially in those with uncorrected, palliated, complex or cyanotic heart conditions, with heart failure the most common cause of premature death.^11^

There is a lack of evidence to inform clinical decision-making in children and adults with CHD. The Cochrane Library contains fewer than 20 reviews on CHD topics,^12^ and there is a need for high-quality, multicentre clinical trials that answer important questions to improve their daily lives and outcomes.^13^ The British Heart Foundation (BHF) identified that ‘we urgently need research breakthroughs to ensure survivors (of CHD) lead longer and healthier lives’.^14^

The James Lind Alliance (JLA) is a non-profit-making initiative, established in 2004 and coordinated by the National Institute for Health and Care Research (NIHR), to bring together patients, carers and clinicians as equals in a Priority Setting Partnership (PSP).^15^ Through a defined method with shared decision making,^16^ PSPs identify and prioritise unanswered questions for research, or evidence uncertainties, providing a platform for researchers and funders to prioritise the research that matters most to research beneficiaries. We, therefore, brought together patients with lived experience of CHD, parents, national charities and healthcare professionals in the UK to form the national Congenital Heart Disease PSP.

## Methods

This process was conducted with reference to the JLA Guidebook (V.10, March 2021)^16^ and reported in accordance with the REPRISE guideline (see online supplemental materials).^17^ ^18^ A steering group of stakeholders with a wide range of lived experiences or professional interests in CHD, including patients, parents, charities and healthcare professionals was formed (see the Acknowledgements section) and agreed a protocol (see online supplemental materials). The scope of the PSP was collectively defined as: ‘The management of CHD throughout life, including prior to birth, focusing on:

10.1136/openhrt-2022-002147.supp1 Supplementary data

- Diagnosis, during pregnancy or after birth.
- Treatment (medical therapy, catheter intervention, surgery including mechanical support and transplantation, lifestyle or psychosocial intervention).
- Outcomes of the conditions and/or treatments and the impact on patients and their families, including the physical, psychological and social effects of living with CHD.’

The PSP excluded from its scope questions about non-management-related aspects of CHD, such as aetiology or non-clinical genetics, acquired heart disease, other than occurring in the context of CHD, and other comorbidities, such as non-cardiac aspects of associated syndromes.

We worked with national charities and professional bodies throughout the process, including survey dissemination and workshop recruitment (see the acknowledgements section). The project was funded by a philanthropic donation, and the funder had no influence on determining the final list of priorities. The steering group anticipated that parents may be the most engaged group, as found in a previous national prioritisation exercise^19^ and noted that there are more healthcare specialists working with children with CHD than with adults. To protect potential priorities for the growing population of adults living with CHD, we, therefore, split the process into parallel child/antenatal and adult tracks at the prioritisation stage. The process comprised four stages, namely: (1) initial survey to gather uncertainties; (2) data processing and evidence checking; (3) interim prioritisation surveys and (4) final priority setting workshops. The steering group met regularly via Zoom video conferencing to oversee the process.

### Initial survey

A cross-sectional, self-administered, public survey was conducted using REDCap. Respondents were asked: ‘What questions would you like to see answered by future research, relating to the diagnosis, treatment or outcomes of congenital heart disease?’ and invited to pose up to three questions. To understand the profile of respondents and any gaps, demographic data were collected on age, gender, ethnicity and role: patient, parent, other relative, healthcare professional, charity, or other. Respondents were asked to provide their name and email address to allow recontact. A link to the online survey was disseminated by our partner organisations via email, newsletters and social media, and promoted via a Facebook page (congenitalPSP) and twitter handle (@congenitalPSP) using the hashtag #CHDpriorities. The project website (www.birmingham.ac.uk/congenital-psp) contained detailed information about the partnership, process, partners and steering group.

The steering group recognised that South Asian and Black communities in the UK are disproportionately affected by CHD,^20^ with double the incidence of severe and complex defects associated with a high infant mortality, compared with the White population.^21^ The proportion unable to ‘speak English well’ is higher among those of South Asian ancestry (Asian-Indian 7.4%, Asian-Pakistani 11.2%, Asian-Bangladeshi 16.2%) than those of Black ancestry (Black-Caribbean 0.3%, Black-African 3.8%).^22^ To facilitate engagement with this population, the survey was translated into Urdu, Bengali, Gujarati and Hindi, with input from the Centre for Ethnic Health Research, along with English, Welsh and Polish, the most commonly spoken immigrant language in the UK,^22^ and made available to download, print and return via a Freepost address. Posters in five languages with a quick response (QR) code link to the online survey were distributed via community networks and mosques, and the printed survey was handed out to targeted demographics during clinic appointments. The PSP Lead also appeared on Living the Life on the Islam Channel, a prime-time contemporary lifestyle television show, to promote the survey among Muslim communities.

### Data processing and evidence checking

Anonymised responses were collated and tagged as relevant to children, adults or both. Submissions identified as out of scope by the steering group were removed. The remaining questions were aggregated into project-specific categories devised by the Information Specialist, in consultation with the PSP Lead. Indicative summary questions were formed from these categories by steering group members, working in clinician/non-clinician pairs (following practice sessions facilitated online during two steering group meetings); using an iterative process, similar or overlapping questions were combined to form summary questions and reworded into plain, consistent language. The steering group reviewed and approved the final list of questions.

A literature search was conducted to identify guidelines and systematic reviews published within the last 5 years (see online supplemental materials), to check whether summary questions had been fully or partially answered. They were considered to be unanswered if a there was no definitive study or systematic review evidence, or recent guidelines concluded that there was insufficient evidence. The resulting longlist of confirmed uncertainties were taken forward to one or both interim prioritisation surveys.

### Interim prioritisation surveys

Two second surveys, one child/antenatal and one adult, were conducted in English using Survey Monkey, which allowed uncertainties to be presented in a random order, to avoid preferencing of questions at the top. A link to the online survey was disseminated through partner organisations and directly to those who had provided an email address in the initial survey. Respondents were asked to select all questions that were important to them, and from these, to choose up to 10 of the most important. Demographic data were collected, and respondents were asked to provide their name, email address and reasons if interested in taking part in a workshop. Completed responses were collated and ranked according to frequency of prioritisation by clinicians and non-clinicians, and the highest ranked questions by each group were taken forward.

### Final priority setting workshops

Two workshops were conducted, one child/antenatal and one adult, involving patients, parents, charities and healthcare professionals. Recruitment used a targeted phased approach through survey respondents and partner organisations to seek non-clinician participation with a range of conditions/backgrounds and clinical representation with a breadth of CHD expertise from centres across the UK and Ireland, aiming for equal numbers of each. Participants were provided with the shortlist of uncertainties and a glossary (see online supplemental materials) in advance. Three experienced JLA advisors facilitated the discussions to build consensus towards the final top 10 priorities using an adapted nominal group technique.^16^ Participants were divided into three small groups, each containing a mix of roles, experience and location, and ensuring that patients/parents were not grouped with their direct healthcare providers. Groups worked together through three discussion and ranking exercises, interspersed with feedback from the other groups, with a whole group session to agree a final ranking, including the top 10 list for each workshop. All participants were reimbursed for travel expenses and patient/parents were paid an honorarium.

## Results

The initial online steering group meeting was held on 16 March 2021, with 12 further online meetings held approximately every 6 weeks throughout the process.

### Initial survey

The initial survey was open from 29 June 2021 to 15 October 2021. A total of 524 individuals completed the survey, of whom 387 (73.9%) were patients or family members (tables 1–2). Most responses were submitted online, with one paper response in English via the Freepost address but none using the translated surveys.

**Table 1:** Roles of survey respondents and workshop participants

Role, n (%)	Initial survey (n=524)	Child/antenatal prioritisation survey (n=250)	Adult prioritisation survey (n=252)	Workshop (n=39)*
Patients with CHD	133 (25.4)	36 (14.4)	151 (59.9)	9 (23)
<16 years	24 (4.5)	18 (7.2)	0	0
≥16 years	109 (20.8)	18 (7.2)	151 (59.9)	9 (23)
Parents	247 (47.1)	105 (42.0)	28 (11.1)	8 (21)
Mothers	227 (43.3)	95 (38.0)	25 (9.9)	7 (18)
Fathers	20 (3.8)	10 (4.0)	3 (1.2)	1 (3)
Other family members	7 (1.3)	5 (2.0)	3 (1.2)	0
Charities†	0	3 (1.2)	2 (0.8)	1 (3)
Healthcare professionals	135 (25.8)	101 (40.4)	68 (27.0)	21 (54)
Paediatric/fetal cardiologists	21 (4.0)	29 (11.6)	5 (2.0)	3 (8)
Adult congenital cardiologists	18 (3.4)	1 (0.4)	27 (10.7)	5 (13)
Congenital cardiac surgeons	21 (4.0)	10 (4.0)	9 (3.6)	6 (15)
Paediatricians/neonatologists with expertise in cardiology	5 (1.0)	7 (2.8)	0	0
Cardiac anaesthetists	7 (1.3)	5 (2.0)	1 (0.4)	0
Paediatric/adult Intensivists	11 (2.1)	17 (6.8)	2 (0.8)	0
Paediatric nurses	18 (3.4)	14 (5.6)	1 (0.4)	3 (8)
Adult congenital nurses	7 (1.3)	0	8 (3.2)	1 (3)
Clinical perfusionists	5 (1.0)	6 (2.4)	4 (1.6)	1 (3)
Other	22 (4.4)‡	12 (4.8)§	7 (2.8)¶	2 (5)**
Unknown	2 (0.4)	–	–	–

*Eleven confirmed participants were unable to attend: 4 parents, 2 patients, 1 adult congenital nurse, 1 clinical psychologist, 1 neonatologist with expertise in cardiology, 1 paediatric intensivist, 1 physiotherapist.

†Some charities responded or attended in a personal capacity as parents or patients.

‡Twenty-two respondents: 4 CHD researchers, 4 physiotherapists, 3 cardiac physiologists, 2 clinical psychologists, 2 echocardiographers, 1 adult cardiac surgeon, 1 adult cardiologist, 1 cardiac pharmacist, 1 general practitioner, 1 paediatric dentist, 1 paediatric dietician, 1 radiologist.

§Twelve respondents: 3 paediatric dieticians, 2 cardiac physiologists, 2 echocardiographers, 1 clinical scientist, 1 general practitioner, 1 obstetrician, 1 paediatric pharmacist, 1 surgical care practitioner.

¶Seven respondents: 4 adult cardiologists, 2 physiotherapists, 1 cardiac physiologist, 1 clinical psychologist, 1 echocardiographer, 1 general practitioner, 1 pulmonary hypertension pulmonologist.

**Two participants: 1 clinical psychologist, 1 non-ACHD cardiologist.

ACHD, adult congenital heart disease.

**Table 2:** Demographics of patient, parent and other family survey respondents and workshop participants

Demographic, n (%)	Initial survey (n=387)	Child/antenatal prioritisation survey (n=146)	Adult prioritisation survey (n=182)	Workshop (n=17)
Age group				
0–10 years	14 (3.6)	22 (15.1)	0	–
11–15 years	10 (2.6)	4 (2.7)	0	–
16–20 years	17 (4.4)	3 (2.1)	13 (7.1)	4 (24)
21–35 years	159 (41.1)	42 (28.8)	58 (31.9)	5 (29)
36–50 years	127 (32.8)	61 (41.8)	56 (30.8)	5 (29)
51–65 years	53 (13.7)	12 (8.2)	49 (26.9)	3 (18)
65+ years	6 (1.6)	2 (1.4)	6 (3.3)	0
Prefer not to say	1 (0.3)	0	0	–
Gender				
Female	325 (84.0)	121 (82.9)	142 (78.0)	13 (76)
Male	59 (15.2)	25 (17.1)	39 (21.4)	3 (18)
Other	0	0	1 (0.5)	1 (6)
Prefer not to say	3 (0.8)	0	0	–
Ethnicity				
Arab or British Arab	3 (0.8)	1 (0.7)	0	0
Asian or British Asian*	17 (4.4)	4 (2.7)	7 (3.8)	2 (12)
Black or Black British^†^	2 (0.5)	1 (0.7)	2 (1.1)	0
Chinese or British Chinese	2 (0.5)	0	0	0
White British or White Irish	335 (86.6)	128 (87.7)	155 (85.2)	14 (82)
Any other White background	15 (3.9)	8 (5.5)	12 (6.6)	0
Mixed/other	8 (2.1)	3 (2.1)	5 (2.7)	1 (6)
Prefer not to say	5 (1.3)	1 (0.7)	1 (0.5)	–

*Asian: Indian or British Indian, Pakistani or British Pakistani, Bangladeshi or British Bangladeshi, any other Asian background.

†Black: African or Black British African, Caribbean or Black British Caribbean, any other Black background.

### Data analysis

**Figure 1:**
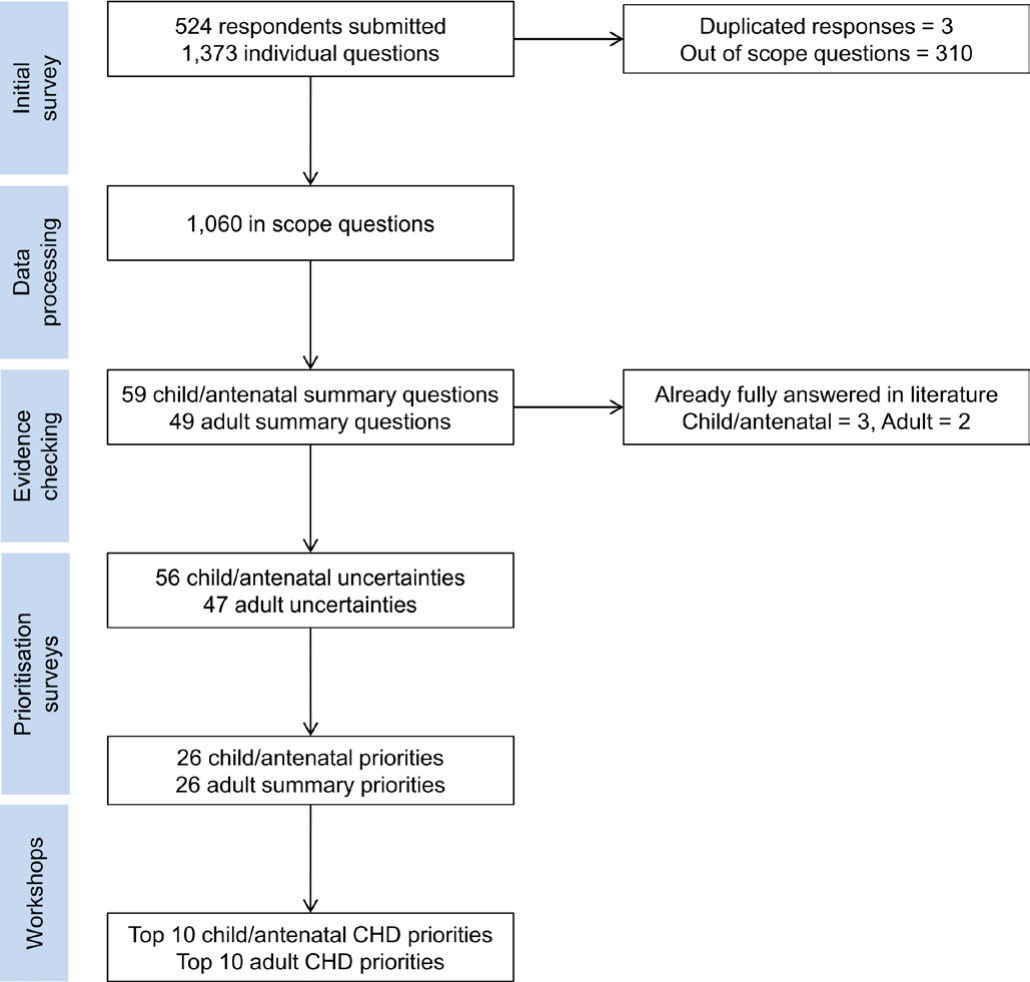
Flow diagram through the Priority Setting Partnership process: from submitted questions, to summary questions, to uncertainties, to priorities. CHD, congenital heart disease.

Respondents submitted 1373 questions, of which 3 were duplicated and 310 were deemed to be out of scope, mostly relating to causes of CHD (figure 1); examples of submitted questions are available on JLA website (https://www.jla.nihr.ac.uk/priority-setting-partnerships/congenital-heart-disease/). The remaining 1060 questions were reviewed by the steering group to generate 59 child/antenatal and 49 adult summary questions. These were individually checked against the existing literature; three child/antenatal and two adult questions were deemed fully answered and removed (see online supplemental materials), leaving 56 child/antenatal and 47 adult uncertainties to progress to the next stage.

### Interim prioritisation surveys

The second surveys were open from 21 March 2022 to 20 May 2022. A total of 446 (85.1%) respondents had provided a valid email address in the initial survey and were contacted directly. A total of 250 respondents completed the child/antenatal survey and 252 completed the adult survey, of whom 146 (58.4%) and 182 (72.2%), respectively, had lived experience of CHD (tables 1 and 2). The full list of questions and interim rankings is provided in online supplemental materials. The top 15 questions ranked by clinicians and/or non-clinicians were taken forward to each workshop, along with two additional questions highly ranked by both groups in the adult survey, providing 26 uncertainties for each workshop. The top ranked questions for young people, fathers and ethnic minority respondents were all included.

### Final priority setting workshops

Workshops were held in person in central Birmingham, UK on 23 June 2022 and 24 June 2022. These were attended by 39 participants: 21 healthcare professionals and 18 non-clinicians, including 9 young people/adults living with CHD, 8 parents of children with CHD and 1 non-patient/parent national charity representative (tables 1 and 2). An additional 11 delegates had agreed to attend but withdrew at short notice due to new COVID-19 infection, family illness or travel disruption caused by national rail strikes. Healthcare professionals with a breadth of interests and expertise from all eleven tertiary CHD programmes in the UK and Ireland were invited, of which nine were represented at one or both workshops (see the Acknowledgements sectoion). There was excellent engagement from all participant groups, with moving personal stories and passionate informed debate; consensus was reached relatively quickly on both days, as shown by the interim small group rankings in online supplemental materials. The final top 10 national research priorities agreed at the workshops are shown in tables 3 and 4.

**Table 3:** Top 10 national priorities for research in child/antenatal congenital heart disease (CHD)

Rank	Priority for research
1	How can damage to organs (eg, heart, brain, lung, kidney, bowel) during heart surgery in children with CHD be minimised to reduce complications, especially in those who require multiple operations?
2	How can prenatal and postnatal screening strategies (eg, scans, pulse oximetry, novel techniques) be improved to achieve greater accuracy, avoid late diagnosis and reduce complications from CHD?
3	What are the effects of CHD, low oxygen saturations and interventions on brain development and behavioural outcomes, and how can these be improved?
4	How can the frequency or need for reoperations be reduced for people with CHD (eg, improved valve/conduit longevity or that grow with the patient)?
5	How can technology be used to deliver personalised care and improve outcomes in CHD (eg, artificial intelligence, 3D printing, genomics, stem cells, organ regeneration)?
6	What is the impact of living with CHD on mental health in children and how can this be improved through access to psychological support and other therapies?
7	What is the impact of living with CHD on quality of life in children and how can this be improved?
8	How can less invasive interventions be performed for CHD with the same outcomes as open-heart surgery?
9	How can the longevity of the Fontan circulation be prolonged and the impact of complications (eg, liver, protein-losing enteropathy, renal, endocrine, fertility) be reduced?
10	What are the long-term outcomes and life expectancy of children born with CHD?

**Table 4:** Top 10 national priorities for research in adult congenital heart disease (CHD)

Rank	Priority for research
1	How can less invasive interventions be performed for CHD with the same outcomes as open-heart surgery?
2	How can the longevity of the Fontan circulation be prolonged and the impact of complications (eg, liver, protein-losing enteropathy, renal, endocrine, fertility) be reduced?
3	What is the impact of living with CHD on mental health in adults and how can this be improved through access to psychological support and other therapies?
4	How can technology be used to deliver personalised care and improve outcomes of those with CHD (eg, artificial intelligence, 3D printing, genomics, stem cells, organ regeneration)?
5	What are the risks and limitations associated with pregnancy, childbirth and motherhood for women with CHD, and what information and support is available?
6	What are the best treatment strategies for heart failure in adults with CHD, in particular those with a systemic right ventricle?
7	How can the management of arrhythmias, including sudden cardiac death, in adults with CHD be improved?
8	How can the indications, timing of referral and outcomes of transplantation and long-term mechanical support in adults with CHD be improved?
9	What is the impact of living with CHD on quality of life in adults and how can this be improved?
10	How can the frequency or need for reoperations be reduced for people with CHD (eg, improved valve/conduit longevity or that grow with the patient)?

## Discussion

Prioritisation of research through consultation with those affected by or caring for those with a disease ensures that it remains directly relevant to improving their lives.^15^ In this endeavour, we established national clinical priorities for research in children and adults with CHD by engaging with patients, parents, national charities and healthcare professionals. Our findings are important because they enable all stakeholders to focus on the questions which matter most to the end users of research and will be publicised widely to inform the direction of CHD research.

CHD is a lifelong condition, and the research priorities identified in this study reflect that journey: from antenatal screening, through reducing the frequency and impact of interventions, to treatments for advanced heart failure and life expectancy. Many encompass holistic outcomes, looking beyond early mortality to improve the quality of survivorship and reduce the impact of living with CHD. They cover a wide range of research areas including surgery, catheter interventions, intensive care, antenatal screening, psychology, cardio-obstetrics, electrophysiology, epidemiology, bioinformatics, pharmacology, technology, bioengineering and transplantation. While diverse methodologies will be required to address these priorities, including qualitative studies, database analysis and translational research, many are well suited to clinical trials. Six priorities on each list are derived from the same summary questions, triangulating and emphasising their importance to both groups, and translating into 14 distinct priorities: 4 child/antenatal-specific, 4 adult-specific and 6 throughout life.

A recent PSP was conducted in adult cardiac surgery for acquired disease^23^ but our PSP is the first time that the JLA process has been applied to CHD, thereby giving patients and their families an equal voice to clinicians.^16^ Several previous studies have identified research priorities in CHD, mostly in adults but with limited patient and public involvement (PPI). Cotts *et al* compiled a list of 45 questions and conducted an international survey of adult CHD providers to identify 10 priority questions, with limited input from patient groups^24^; unsurprisingly, the priorities were more specific and medically focused research questions. Helm *et al* conducted an online survey of adult patients with four specific conditions, their relatives and physicians in Germany to prioritise predetermined research topics.^25^ While the topics were less well defined, many were similar to the priorities we identified, including arrythmias and sudden death, pregnancy, mental health, Fontan failure, reoperations and heart failure. In the USA, Gurvitz *et al* reported the findings of a multidisciplinary working group composed of researchers in adult CHD and related fields, and one PPI representative, ‘recognising the importance of the patient perspective in research efforts’.^26^ Some of the priority topics identified were similar to our PSP, such as mechanical circulatory support/transplantation, sudden cardiac death, late outcomes of single ventricle heart conditions, mental health and pregnancy. Finally, Drury *et al*^19^ conducted a UK national study to identify research priorities in single ventricle heart conditions, involving patients, parents, healthcare professionals, researchers and charities. Priorities included prolonging the longevity of the Fontan circulation, brain development, heart failure, pregnancy, transplantation and use of new technology.

By defining national clinical priorities for research, we intend that this PSP will be transformative for collaborative CHD research in the UK.^27^ A national strategy has been developed to address these priorities, with endorsement from our national charity and professional partners, and includes establishing:

- A UK and Ireland network for conducting multicentre studies, similar to the Paediatric Heart Network,^28^ focusing on clinical trials that address the priorities and have the potential to change clinical practice. It will use existing infrastructure such as the BHF Clinical Research Collaborative, NIHR Clinical Research Networks and established Clinical Trials Units with expertise in multi-centre paediatric, interventional and surgical trials.
- A national CHD PPI group, composed of engaged patient, parent and charity members, who will actively contribute to the development, conduct and reporting of research.
- Working groups focusing on one or more related priorities, comprising clinicians, researchers and PPI members, and overseen by the network. Their role will be to translate the priorities into specific research questions, lead project development and coordinate funding applications. NIHR recognises the importance of the JLA process and have established rolling calls across their funding programmes dedicated to studies that address priorities identified by PSPs.^29^

The strengths of this project include the use of a recognised, equitable and robust process for determining national research priorities, good engagement with stakeholder groups throughout the process, and broad representation of backgrounds, interests and centres at the workshops. A clear consensus between groups emerged rapidly during both workshops and the top 10 have appeal across the clinical spectrum. While there was overlap between the two lists, the top three child/antenatal priorities are paediatric-specific, while the adult-specific priorities are lower down and may have been squeezed out if conducted as a single process. Splitting at the interim prioritisation stage therefore fulfilled its role of protecting adult-specific priorities but may have inadvertently marginalised questions related to transition. While rail strikes disrupted attendance on both days, PPI attendance payments and reimbursement for expenses, in line with national standards, widened access and participation in the workshops.

There were several limitations. We explicitly focused on clinical priorities to maximise the potential for translation into clinical trials with potential patient benefit in the short term to medium term; however, by excluding non-management related uncertainties, such as causes of CHD, we dismissed the major translational impact which these more fundamental questions may have for future generations. We sought responses only from those living in the UK and working in the National Health Service, potentially limiting its international applicability. Despite extensive efforts to engage with ethnic minority communities, there were few responses by those of South Asian or Black ancestry, and only three non-White British/Irish patients or parents attended the workshops; this has been observed in other PSPs^30^ and will be explored in a separate paper. Similarly, fathers were relatively under-represented in both the surveys and workshops.

In conclusion, we brought together patients, their families, charities and healthcare professionals in a PSP to determine UK national priorities for research in CHD using an established shared decision-making process. This partnership provides a unique opportunity for the UK CHD community to develop collaborative research, through a national strategy to address the priorities including a network for multicentre clinical trials, a national PPI group and working groups to translate the most important questions into studies that can have an impact on patient care and improve outcomes.

10.1136/openhrt-2022-002147.supp2 Supplementary data

10.1136/openhrt-2022-002147.supp3 Supplementary data

## Data Availability

Data are available in a public, open access repository. Key documents and datasets are accessible on the JLA website: https://www.jla.nihr.ac.uk/priority-setting-partnerships/congenital-heart-disease/.
