# Supplementary material for: Research priorities in children and adults with congenital heart disease: a James Lind Alliance Priority Setting Partnership
Source: Open Heart. 2022 Nov 22;9(2):e002147. doi: 10.1136/openhrt-2022-002147 (PMC9843188; doi:10.1136/openhrt-2022-002147)
Supplement: Supplementary data [file openhrt-2022-002147supp001.pdf]

SUPPLEMENTARY MATERIALS

Evidence checking process

A literature search was conducted to identify guidelines and systematic reviews published within the last five years using generic CHD text and index terms. The following sample strategy was used and modified as necessary for the different databases, with inclusion of corresponding index terms when available.

1. (heart adj2 (defect* or abnormal* or malform*))
2. (congenital adj2 (heart or cardiac or cardio*))
3. OR/1 to 2

The results were used for the evidence check against the bulk of the questions. When a question referenced a specific condition within the CHD umbrella, a further search was carried out using terms specific to that particular condition.

The following sources were searched:

Clinical guidelines:

- National Institute for Health and Care Excellence (NICE)
- Scottish Intercollegiate Guidelines Network (SIGN)
- PubMed, using filter: guidelines only
- Royal College of Paediatrics and Child Health, European Society of Cardiology, American Heart Association

Systematic Reviews (last 5 years):

- Cochrane Database of Systematic Reviews
- Epistemonikos
- PubMed, using filter: systematic reviews only

We also consulted with experts on the Steering Group to identify key papers or guidance and carried out citation searches.

Drury NE et al. Research priorities in congenital heart disease

Questions deemed fully answered and removed during evidence checking

Question 1. What are the outcomes of heart transplantation across all UK centres? (both)

Evidence: National report on cardiothoracic organ transplantation, including adult and paediatric heart transplantation, with outcomes reported at 30 days, 90 days, 1 year, and 5 years from all 7 UK centres.

Source: NHS Blood and Transplant. Annual report on Cardiothoracic Organ Transplantation. Report for 2019/2020 (1 April 2010 – 31 March 2020). https://www.odt.nhs.uk/statistics-and-reports/organ-specific-reports/ [accessed March 2022].

Question 2. What are the outcomes of staged management of hypoplastic left heart syndrome across all UK centres? (child/antenatal)

Evidence: National study describing the long-term outcomes, treatment pathways and risk factors for patients diagnosed with hypoplastic left heart syndrome (HLHS) born between 2000 and 2015 in England and Wales, using data from the National Congenital Heart Disease Audit. Additional perioperative outcomes from Scotland and Ireland available from NCHDA report.

Sources: Rogers L, Pagel C, Sullivan ID et al. Interventions and Outcomes in Children With Hypoplastic Left Heart Syndrome Born in England and Wales Between 2000 and 2015 Based on the National Congenital Heart Disease Audit. *Circulation* 2017;136(18):1765-7.

National Congenital Heart Disease Audit (NCHDA). https://www.nicor.org.uk/wp-content/uploads/2021/10/NCHDA-Domain-Report_2021_FINAL.pdf [accessed March 2022].

Question 3. What are the outcomes of surgery for CHD across all UK centres? (both)

Evidence: The National Congenital Heart Disease Audit (NCHDA) was set up in 2000 as the Central Cardiac Audit Database (CCAD for Congenital Heart Disease) to assess patient outcomes after therapeutic paediatric and congenital cardiovascular procedures (surgery, transcatheter and electrophysiological interventions) at all centres in the UK and the Republic of Ireland (since 2012) as well as the success of antenatal screening. The audit focuses on monitoring activity levels by compiling outcomes following congenital cardiovascular procedures with the aim to contribute to quality assurance (QA) and development of care.

Source: National Congenital Heart Disease Audit (NCHDA). https://www.nicor.org.uk/wp-content/uploads/2021/10/NCHDA-Domain-Report_2021_FINAL.pdf [accessed March 2022].

Supplementary Table 1. Child/antenatal interim prioritisation survey results.

Rank	Question	HCP rank	Non-HCP rank	Average rank	Combined rank	Progress
1	What are the effects of CHD, low oxygen saturations, and interventions on brain development and behavioural outcomes, and how can these be improved?	5	3	4	1	Yes
2	How can damage to organs (eg. heart, brain, lung, kidney, bowel) during heart surgery in children with CHD be minimised to reduce complications, especially in those who require multiple operations?	2	11	6.5	4	Yes
3	How can pre- and post-natal screening strategies (eg. scans, pulse oximetry, novel techniques) be improved to achieve greater accuracy, avoid late diagnosis, and reduce complications from CHD?	8	12	10	9	Yes
4	What are the long-term outcomes and life expectancy of children born with CHD?	15	7	11	6	Yes
5	How can technology be used to deliver personalised care and improve outcomes in CHD (eg. artificial intelligence, 3D printing, genomics, stem cells, organ regeneration)?	3	20	11.5	11	Yes
6	What is the impact of a child living with CHD on their parents, siblings and wider family, and what support (psychological, social, other) could be provided?	22	2	12	3	Yes
7=	What lifestyle factors (e.g. exercise, diet, or other interventions) may improve health-related outcomes in children with CHD?	19	6	12.5	5	Yes
7=	How can the longevity of the Fontan circulation be prolonged and the impact of complications (eg. liver, protein-losing enteropathy (PLE), renal, endocrine, fertility) be reduced?	6	19	12.5	13	Yes
9=	What is the impact of living with CHD on mental health in children and how can this be improved through access to psychological support and other therapies?	25	1	13	2	Yes
9=	What is the impact of living with CHD on quality of life in children and how can this be improved?	19	7	13	7	Yes
9=	How can the frequency or need for reoperations be reduced for people with CHD (eg. improved valve/conduit longevity or that grow with the patient)?	13	13	13	13	Yes
12	How can antenatal counselling of parents about the diagnosis and implications of CHD be improved?	13	14	13.5	15	Yes

Drury NE et al. Research priorities in congenital heart disease

13	What are the risk factors for complications following surgery for CHD in children and how can these be predicted (eg. genetics, biomarkers)?	3	31	17	17	Yes
14	How can data be shared effectively and used to identify variations in CHD treatment, develop national guidelines and inform best practice (eg. national registries of rare diseases)?	7	29	18	19	Yes
15=	What are the most appropriate anticoagulation strategies for children with different types of CHD?	10	27	18.5	21	Yes
15=	What strategies around the time of surgery can improve short-term outcomes in children following surgery for CHD (eg. goal-directed therapy, enhanced recovery, neuromonitoring, anti-inflammatory drugs)?	1	36	18.5	18	Yes
17	What are the best approaches to feeding in babies with CHD, to optimise growth, improve outcomes, and reduce parental distress?	15	25	20	22	Yes
18	How can less invasive interventions be performed for CHD with the same outcomes as open-heart surgery?	32	10	21	12	Yes
19=	How can the timing of surgery for CHD be improved to achieve better long-term outcomes?	25	18	21.5	19	
19=	What is the role of remote monitoring in CHD to reduce complications and optimise timing of interventions (eg. connected devices, home monitors, mobile apps)?	11	32	21.5	24	Yes
21	What new procedures could offer better long-term outcomes than the Fontan circulation, including for those unsuitable for Fontan completion?	22	23	22.5	24	-
22=	How can GPs and other healthcare professionals be better informed about CHD?	41	5	23	8	Yes
22=	What are the best ways to support mothers and babies with CHD in bonding and establishing/continuing breastfeeding, before and after surgery?	25	21	23	23	-
24	How does the perception of themselves, their condition and treatments change in children with CHD as they become older, what influences this and how does it impact on their future healthcare?	43	4	23.5	9	Yes
25	What is the role of genetic screening in people with CHD, their siblings and other family members?	25	23	24	28	
26=	What are the best treatment strategies for heart failure in children with CHD, in particular those with a systemic right ventricle?	17	35	26	31	-

26=	What are the barriers to conducting multi-centre clinical trials in CHD and how can they be overcome?	11	41	26	31	Yes
28	What are the indications for extra-corporeal life support (ECLS) following surgery for CHD, how effective is it as a bridge to recovery, and what are the long-lasting effects?	8	48	28	35	Yes
29=	How can children with CHD, their families, schools, and the wider public be better informed about the impact of living with CHD?	48	9	28.5	15	Yes
29=	What is the impact of CHD on educational and employment attainments and how can these be improved?	43	14	28.5	24	Yes
29=	What other health conditions are associated with CHD and how can these be best managed?	43	14	28.5	24	Yes
32	How can paediatric-specific devices for CHD (eg. catheter technology, long-term mechanical support) be better developed and introduced to improve outcomes?	25	34	29.5	34	-
33=	How can the communication and understanding of risks associated with CHD be improved?	36	25	30.5	29	-
33=	What are the most important palliative care and end of life care needs for people with CHD and their families and how can these best be delivered?	17	44	30.5	38	-
35	How can the development of pulmonary hypertension in children with CHD be predicted, prevented, diagnosed, and treated?	25	37	31	40	-
36	How can the decision-making be improved on which patients would benefit from biventricular repair versus single ventricle pathway?	21	43	32	40	-
37	How can holistic, integrated care for patients with CHD and their families be best provided across all stages, including transition to adult services?	41	27	34	33	-
38=	What is the relationship between oral/dental care and long-term cardiovascular health in people with CHD?	53	17	35	30	-
38=	How can the management of arrhythmias, including sudden cardiac death, in children with CHD be improved?	37	33	35	37	-
40	What is the impact of dedicated, centralised programmes for the management of patients with complex CHD (eg. functionally single ventricle) from diagnosis through adulthood, on clinical outcomes and health economics?	22	51	36.5	43	-
41	What are the causes and potential treatments for leg pain in children with CHD?	54	21	37.5	35	-

42	What are the best ways to prevent and treat endocarditis in children with different types of CHD and interventions?	47	29	38	38	-
43	What is the role of prenatal interventions in improving the outcomes of those with CHD (eg. oxygen therapy, invasive catheter/surgical procedures)?	31	46	38.5	42	-
44	How can the indications, timing of referral, and outcomes of transplantation and long-term mechanical support in children with CHD be improved?	32	46	39	43	-
45	What are the functional outcomes of children who sustain morbidities following surgery for CHD?	32	48	40	45	-
46	How can the diagnosis, treatment and outcomes of inherited cardiomyopathies be improved?	32	51	41.5	46	-
47	What is the role of physiotherapy in the management and well-being of children with CHD?	48	37	42.5	47	-
48	Do classifications of CHD based on heart function better predict health related outcomes, compared to conventional classifications based on anatomy?	43	44	43.5	48	-
49=	What is the optimal treatment and timing of intervention in people with Ebstein anomaly?	40	48	44	48	-
49=	What demographic factors (eg. race, sex, socioeconomic) affect the incidence and outcomes of CHD?	37	51	44	48	-
51=	How can implantable pacemakers for CHD be improved to reduce need for re-intervention (eg. durability, battery life)?	52	37	44.5	48	-
51=	What is the best routine follow-up protocol for people with a Fontan circulation?	48	41	44.5	48	-
53	What is the impact of COVID-19 on children with CHD, their access to services and outcomes?	54	37	45.5	53	-
54	What are the best treatment strategies for preterm babies with a large persistent arterial duct?	37	56	46.5	54	-
55	What is the role of fenestration in the management of children with a Fontan circulation?	48	54	51	55	-
56	How can the use of immunosuppressive drugs following heart transplant for CHD be improved?	54	55	54.5	56	-

Blue indicates group ranking 1-15, progressed to the workshop.

Drury NE et al. Research priorities in congenital heart disease

Supplementary Table 2. Adult interim prioritisation survey results.

Rank	Question	HCP rank	Non-HCP rank	Average rank	Combined rank	Progress
1	How can the management of arrhythmias, including sudden cardiac death, in adults with CHD be improved?	2	9	5.5	8	Yes
2=	How can technology be used to deliver personalised care and improve outcomes of those with CHD (eg. artificial intelligence, 3D printing, genomics, stem cells, organ regeneration)?	8	7	7.5	7	Yes
2=	What are the risks and limitations associated with pregnancy, childbirth, and motherhood for women with CHD, and what information and support is available?	4	11	7.5	9	Yes
4	What is the impact of living with CHD on mental health in adults and how can this be improved through access to psychological support and other therapies?	15	1	8	1	Yes
5	What lifestyle factors (e.g. exercise, diet, or other interventions) may improve health-related outcomes in adults with CHD?	12	6	9	4	Yes
6=	What are the long-term outcomes and life expectancy of adults with CHD?	21	2	11.5	2	Yes
6=	How can the longevity of the Fontan circulation be prolonged and the impact of complications (eg. liver, protein-losing enteropathy (PLE), renal, endocrine, fertility) be reduced?	2	21	11.5	13	Yes
8=	How can GPs and other healthcare professionals be better informed about CHD?	21	4	12.5	5	Yes
8=	What are the most appropriate anticoagulation strategies for adults with different types of CHD?	12	13	12.5	14	Yes
10	What are the best treatment strategies for heart failure in adults with CHD, in particular those with a systemic right ventricle?	1	29	15	10	Yes
11=	What is the impact of living with CHD on quality of life in adults and how can this be improved?	26	5	15.5	6	Yes
11=	What is the optimal timing for pulmonary valve replacement in those with pulmonary regurgitation late after complete repair of tetralogy of Fallot?	8	23	15.5	15	Yes
13	What is the role of remote monitoring in CHD to reduce complications and optimise timing of interventions (eg. connected devices, home monitors, mobile apps)?	16	17	16.5	15	Yes *

Drury NE et al. Research priorities in congenital heart disease

14	How can the frequency or need for reoperations be reduced for people with CHD (eg. improved valve/conduit longevity or that grow with the patient)?	17	17	17	19	Yes *
15	What is the role of genetic screening in people with CHD, their siblings and other family members?	20	15	17.5	15	Yes
16	What other health conditions are associated with CHD later in life and how can the risk of these be best managed?	34	3	18.5	3	Yes
17	What are the late outcomes for adults living with a Fontan circulation and what is the role of transplantation and its alternatives, including novel therapies?	4	34	19	19	Yes
18	How can data be shared effectively and used to identify variations in CHD treatment, develop national guidelines and inform best practice (eg. national registries of rare diseases)?	12	27	19.5	21	Yes
19	What strategies around the time of surgery can improve short-term outcomes in adults following surgery for CHD (eg. prehabilitation, enhanced recovery, transfusion, physiotherapy)?	17	23	20	23	-
20	What are the effects of CHD, low oxygen saturations, and interventions on brain development and behavioural outcomes, and how can these be improved?	29	12	20.5	15	Yes
21	How can the timing of surgery for CHD be improved to achieve better long-term outcomes?	26	16	21	21	-
22=	How can less invasive interventions be performed for CHD with the same outcomes as open-heart surgery?	34	10	22	12	Yes
22=	How can the indications, timing of referral, and outcomes of transplantation and long-term mechanical support in adults with CHD be improved?	4	40	22	27	Yes
24	What are the most important palliative care and end of life care needs for people with CHD and their families and how can these best be delivered?	10	35	22.5	25	Yes
25=	How can adults with CHD, their families, employers, and the wider public be better informed about the impact of living with CHD?	41	8	24.5	11	Yes
25=	How can the development of pulmonary hypertension in adults with CHD be predicted, prevented, diagnosed, and treated?	21	28	24.5	27	-
25=	What is the impact of dedicated, centralised programmes for the management of patients with complex CHD (eg. functionally single ventricle) from diagnosis through adulthood, on clinical outcomes and health economics?	7	42	24.5	33	Yes

28	How can the long-term outcomes of people with CHD who had now historical procedures be improved (eg. Mustard operation)?	21	29	25	29	-
29	What are the best ways to prevent and treat endocarditis in adults with different types of CHD and interventions?	38	14	26	23	Yes
30	What are the barriers to conducting multi-centre clinical trials in CHD and how can they be overcome?	10	44	27	39	Yes
31	What is the impact of CHD on educational and employment attainments and how can these be improved?	38	17	27.5	26	-
32	What is the best way to treat adults with CHD diagnosed later in life?	29	29	29	30	-
33=	What is the best routine follow-up protocol for people with a Fontan circulation?	26	35	30.5	31	-
33=	What is the relationship between oral/dental care and long-term cardiovascular health in people with CHD?	44	17	30.5	31	-
35=	How can implantable pacemakers for CHD be improved to reduce need for re-intervention (eg. durability, battery life)?	41	23	32	33	-
35=	What is the optimal treatment and timing of intervention in people with Ebstein anomaly?	17	47	32	45	-
37	What information and support can be provided to reduce social isolation in people with CHD?	43	23	33	36	-
38=	How can the communication and understanding of risks associated with CHD be improved?	32	35	33.5	33	-
38=	Should people with an abnormal course of a coronary artery, passing between the two main arteries but without any symptoms, be operated on?	21	46	33.5	45	-
40=	How can holistic, integrated care for patients with CHD and their families be best provided across all stages, including transition to adult services?	40	29	34.5	37	-
40=	What is the impact of COVID-19 on adults with CHD, access to services and outcomes?	47	22	34.5	37	-
42=	What is the role of physiotherapy in the management and well-being of adults with CHD?	34	38	36	39	-
42=	What demographic factors (eg. race, sex, socioeconomic) affect the incidence and outcomes of CHD?	29	43	36	44	-

44=	Do classifications of CHD based on heart function better predict health related outcomes, compared to conventional classifications based on anatomy?	34	39	36.5	42	-
44=	What are the late consequences of coarctation of the aorta repaired during childhood and how can adult outcomes be improved?	32	41	36.5	43	-
46	What are the causes and potential treatments for leg pain in adults with CHD?	44	33	38.5	41	-
47	How can the use of immunosuppressive drugs following heart transplant for CHD be improved?	44	45	44.5	47	-

Blue indicates group ranking 1-15, progressed to the workshop. * indicates progression based on high combined ranking.

Drury NE et al. Research priorities in congenital heart disease

Supplementary Table 3. Child/antenatal workshop prioritisation by small groups, first round.

Rank	Question (abbreviated)	G1	G2	G3	Combined
1	Minimise organ damage during surgery to reduce complications, especially in multiple operations	1	1	3	5
2	Pre- and post-natal screening strategies to improve accuracy, avoid late diagnosis, and reduce complications	5	3	2	10
3	Effects of CHD, low oxygen saturations, and interventions on brain development and behaviour	8	2	4	14
4	Reduce the frequency or need for reoperations	10	5	1	16
5	Impact of living with CHD on mental health and access to psychological support and other therapies	3	4	14	21
6	Technology to deliver personalised care and improve outcomes	2	13	10	25
7	Less invasive interventions with same outcomes as open-heart surgery	9	16	5	30
8	Long-term outcomes and life expectancy of children born with CHD	17	7	7	31
9	Strategies around the time of surgery can improve short-term outcomes	4	11	16	31
10	Impact on quality of life in children and how to improve	16	10	6	32
11	Prolong longevity of the Fontan circulation and reduce complications	18	6	11	35
12	Remote monitoring to reduce complications and optimise timing of interventions	7	8	23	38
13	Children's perception changes as they become older	19	14	8	41
14	Data sharing to identify variations in care, develop guidelines and inform practice	13	17	15	45
15	Impact on parents, siblings and wider family, and provision of support	11	25	9	45
16	Lifestyle factors to improve health-related outcomes	24	12	12	48
17	Antenatal counselling of parents about diagnosis and implications	6	24	19	49
18	Risk factors for complications following surgery	12	20	17	49
19	Make everyone better informed about the impact of living with CHD	21	9	21	51
20	Extra-corporeal life support (ECLS) following surgery, indications, bridge to recovery, and long-lasting effects	22	23	13	58
21	Feeding in babies with CHD, to optimise growth, improve outcomes, and reduce parental distress	15	19	25	59
22	Impact on educational and employment attainments	20	21	18	59
23	Informing GPs and other healthcare professionals about CHD	25	15	20	60
24	Anticoagulation strategies for children with different types of CHD	23	18	22	63
25	Other health conditions associated with CHD and their management	14	26	26	66
26	Overcoming barriers to multi-centre clinical trials	26	22	24	72

Blue indicates group ranking 1-10, yellow 11-20, red 21-26. G, small group; GP, general practitioner.

Drury NE et al. Research priorities in congenital heart disease

Supplementary Table 4. Child/antenatal workshop prioritisation by small groups, second round.

Rank	Question (abbreviated)	G1	G2	G3	Combined
1	Minimise organ damage during surgery to reduce complications, especially in multiple operations	1	1	1	3
2	Pre- and post-natal screening strategies to improve accuracy, avoid late diagnosis, and reduce complications	2	3	2	7
3	Effects of CHD, low oxygen saturations, and interventions on brain development and behaviour	3	2	3	8
4	Reduce the frequency or need for reoperations	5	4	4	13
5	Technology to deliver personalised care and improve outcomes	6	6	6	18
6	Impact of living with CHD on mental health and access to psychological support and other therapies	4	5	11	20
7	Impact on quality of life in children and how to improve	9	10	5	24
8	Less invasive interventions with same outcomes as open-heart surgery	7	11	7	25
9	Prolong longevity of the Fontan circulation and reduce complications	10	7	8	25
10	Strategies around the time of surgery can improve short-term outcomes	11	9	10	30
11	Long-term outcomes and life expectancy of children born with CHD	15	8	9	32
12	Remote monitoring to reduce complications and optimise timing of interventions	13	12	14	39
13	Impact on parents, siblings and wider family, and provision of support	8	15	16	39
14	Data sharing to identify variations in care, develop guidelines and inform practice	16	14	12	42
15	Children's perception changes as they become older	14	13	15	42
16	Lifestyle factors to improve health-related outcomes	12	16	17	45
17	Risk factors for complications following surgery	18	18	13	49
18	Antenatal counselling of parents about diagnosis and implications	17	17	18	52
19	Make everyone better informed about the impact of living with CHD	19	19	19	57
20	Extra-corporeal life support (ECLS) following surgery, indications, bridge to recovery, and long-lasting effects	20	20	20	60
21	Feeding in babies with CHD, to optimise growth, improve outcomes, and reduce parental distress	21	21	21	63
22	Impact on educational and employment attainments	22	22	22	66
23	Informing GPs and other healthcare professionals about CHD	23	23	23	69
24	Anticoagulation strategies for children with different types of CHD	24	24	24	72
25	Other health conditions associated with CHD and their management	25	25	25	75
26	Overcoming barriers to multi-centre clinical trials	26	26	26	78

Blue indicates group ranking 1-10, yellow 11-20, red 21-26. G, small group; GP, general practitioner.

Drury NE, *et al. Open Heart* 2022; 9:e002147. doi: 10.1136/openhrt-2022-002147

Drury NE et al. Research priorities in congenital heart disease

Supplementary Table 5. Adult workshop prioritisation by small groups, first round.

Rank	Question (abbreviated)	G1	G2	G3	Combined
1	Less invasive interventions with same outcomes as open-heart surgery	3	4	6	13
2	Prolong longevity of the Fontan circulation and reduce complications	12	3	2	17
3	Technology to deliver personalised care and improve outcomes	10	5	3	18
4	Indications, timing, and outcomes of transplantation and long-term mechanical support	4	8	7	19
5	Pregnancy, childbirth, and motherhood for women with CHD, information and support available	6	10	8	24
6	Impact of living with CHD on mental health and access to psychological support and other therapies	1	9	15	25
7	Management of arrhythmias, including sudden cardiac death	11	11	4	26
8	Treatment strategies for heart failure, in particular systemic right ventricle	21	6	1	28
9	Reduce the frequency or need for reoperations	16	1	13	30
10	Impact on quality of life in adults and how to improve	8	18	5	31
11	Long-term outcomes and life expectancy of adults with CHD	2	17	14	33
12	Timing of pulmonary valve replacement late after complete repair of tetralogy of Fallot	17	7	12	36
13	Late outcomes of Fontan circulation and role of transplantation and alternatives	5	14	17	36
14	Other health conditions associated with CHD later in life and their management	13	2	23	38
15	Data sharing to identify variations in care, develop guidelines and inform practice	9	21	9	39
16	Anticoagulation strategies for adults with different types of CHD	24	13	10	47
17	Lifestyle factors to improve health-related outcomes	23	15	11	49
18	Overcoming barriers to multi-centre clinical trials	7	26	19	52
19	Palliative care and end of life care needs and how best delivered	20	19	16	55
20	Make everyone better informed about the impact of living with CHD	15	16	25	56
21	Prevention and treatment of endocarditis	25	12	20	57
22	Effects of CHD, low oxygen saturations, and interventions on brain development and behaviour	14	24	21	59
23	Remote monitoring to reduce complications and optimise timing of interventions	18	20	22	60
24	Informing GPs and other healthcare professionals about CHD	19	22	26	67
25	Role of genetic screening in people with CHD, their siblings and other family members	26	23	18	67
26	Dedicated, centralised programmes for complex CHD from diagnosis through adulthood	22	25	24	71

Blue indicates group ranking 1-10, yellow 11-20, red 21-26. G, small group; GP, general practitioner.

Supplementary Table 6. Adult workshop prioritisation by small groups, second round.

Rank	Question (abbreviated)	G1	G2	G3	Combined
1	Less invasive interventions with same outcomes as open-heart surgery	4	1	1	6
2	Prolong longevity of the Fontan circulation and reduce complications	3	3	2	8
3	Technology to deliver personalised care and improve outcomes	5	4	3	12
4	Pregnancy, childbirth, and motherhood for women with CHD, information and support available	2	9	6	17
5	Treatment strategies for heart failure, in particular systemic right ventricle	8	7	4	19
6	Impact of living with CHD on mental health and access to psychological support and other therapies	1	2	17	20
7	Management of arrhythmias, including sudden cardiac death	7	6	8	21
8	Indications, timing, and outcomes of transplantation and long-term mechanical support	6	5	10	21
9	Impact on quality of life in adults and how to improve	10	10	5	25
10	Reduce the frequency or need for reoperations	9	8	11	28
11	Timing of pulmonary valve replacement late after complete repair of tetralogy of Fallot	13	12	7	32
12	Long-term outcomes and life expectancy of adults with CHD	11	11	12	34
13	Overcoming barriers to multi-centre clinical trials	12	19	9	40
14	Late outcomes of Fontan circulation and role of transplantation and alternatives	14	14	13	41
15	Anticoagulation strategies for adults with different types of CHD	17	13	14	44
16	Lifestyle factors to improve health-related outcomes	15	18	15	48
17	Other health conditions associated with CHD later in life and their management	18	15	16	49
18	Data sharing to identify variations in care, develop guidelines and inform practice	16	17	18	51
19	Palliative care and end of life care needs and how best delivered	19	20	19	58
20	Prevention and treatment of endocarditis	21	16	21	58
21	Make everyone better informed about the impact of living with CHD	20	21	20	61
22	Effects of CHD, low oxygen saturations, and interventions on brain development and behaviour	22	22	22	66
23	Remote monitoring to reduce complications and optimise timing of interventions	23	23	23	69
24	Informing GPs and other healthcare professionals about CHD	24	24	24	72
25	Role of genetic screening in people with CHD, their siblings and other family members	25	25	25	75
26	Dedicated, centralised programmes for complex CHD from diagnosis through adulthood	26	26	26	78

Blue indicates group ranking 1-10, yellow 11-20, red 21-26. G, small group; GP, general practitioner.

Plain English glossary of terms used in the lists of questions at the workshops, child/antenatal and adult combined

Antenatal/pre-natal	Before birth, during or relating to pregnancy.
Anticoagulation	Treatment with drugs to prevent and treat blood clots in blood vessels and the heart.
Anti-inflammatory drugs	Medications that reduce inflammation (redness, swelling, pain) in the body, e.g. steroids, ibuprofen.
Arrhythmias	Conditions in which the heart beats with an irregular or abnormal rhythm.
Artificial intelligence	The theory and development of computer systems able to perform tasks normally requiring human intelligence, such as visual perception, speech recognition, and decision-making.
Biomarkers	Biological molecules found in blood, other body fluids, or tissues that are a sign of a normal or abnormal process, or of a condition or disease.
Bridge to recovery	Use of a short-term therapy to allow an organ e.g. the heart, to recover sufficient function to no longer require such support.
Clinical trials	A type of research study that tests how well new medical approaches work in people, such as new methods of screening, prevention, diagnosis, or treatment of a disease.
Conduit	A tube used to allow the flow of blood between two structures, e.g. right ventricle to pulmonary artery; may or may not contain a valve.
Endocarditis	A rare and life-threatening condition where the inner lining of the heart chambers and/or valves becomes inflamed, often due to an infection e.g. bacteria, leading to structural damage.
Enhanced recovery	Patient-centred approach that helps people recover more quickly after having major surgery.
Extra-corporeal life support (ECLS)	A technique using a machine to provide short-term support for the heart and lungs, in theatre and/or intensive care, where the blood passes outside of the body to have oxygen added and is pumped back into the body.

Fontan circulation	The surgically constructed pathway for blood returning from the body to go to the lungs without being pumped by the heart. Used as a treatment in children who lack two fully developed pumping chambers (ventricles), often as a third-stage operation. Named after Francis Fontan (1929-2018), surgeon.
Functionally single ventricle	A term used to describe congenital heart defects which lack two fully developed pumping chambers (ventricles) e.g. hypoplastic left heart syndrome, tricuspid atresia, double inlet left ventricle.
Genetic screening	The study of a person's DNA to identify genetic differences or susceptibility to particular diseases or abnormalities.
Genomics	The study of the complete set of DNA (including all of its genes) in a person or other organism.
Goal-directed therapy	An approach using monitoring techniques to guide the administration of different treatments e.g. fluids and drugs to support the heart and circulation on intensive care.
Mechanical support	A treatment for severe heart failure using a machine to support the function of the heart, most commonly a ventricular assist device (VAD), either inside or outside of the body.
Neuromonitoring	Techniques for monitoring the brain, spinal cord and/or other nerves e.g. measuring brain waves.
Organ regeneration	The process of implanting or integrating man-made materials into a human to replace natural organs or tissues e.g. growing a new heart.
Oxygen saturations	The percentage of oxygen in a person's blood.
Post-natal	Relating to or denoting the period after childbirth.
Protein-losing enteropathy (PLE)	A condition in which proteins leak into the gut, leading to low protein levels in the blood; may be associated with the Fontan circulation.
Pulmonary regurgitation	A condition in which the pulmonary valve, which controls blood flow from the right side of the heart to the lungs, does not close properly and a portion of the blood leaks back into the right side of the heart; occurs commonly after complete repair of tetralogy of Fallot.
Pulse oximetry	A non-invasive technique for measuring oxygen saturations, usually with a probe on a finger or ear.

Stems cells	Cells from which other types of cells with specialist functions can develop e.g. heart cells.
Systemic right ventricle	A group of conditions in which the pumping chamber (ventricle) that supplies blood to the body is, or has the appearance of, the pumping chamber that would usually supply blood to the lungs, e.g. transposition following atrial switch operation (Mustard, Senning), congenitally corrected transposition, and hypoplastic left heart syndrome following Fontan completion.
Tetralogy of Fallot	A type of congenital heart defect consisting of amongst others, a hole between the pumping chambers (ventricular septal defect) and narrowing of the exit from the right ventricle to the lungs (pulmonary stenosis), leading to blue blood going round the body rather than to the lungs; hence the term ‘blue baby’. Named after Étienne-Louis Arthur Fallot (1850-1911), physician.
