# Supplementary material for: Research priorities in children and adults with congenital heart disease: a James Lind Alliance Priority Setting Partnership
Source: Open Heart. 2022 Nov 22;9(2):e002147. doi: 10.1136/openhrt-2022-002147 (PMC9843188; doi:10.1136/openhrt-2022-002147)
Supplement: Supplementary data [file openhrt-2022-002147supp002.pdf]

James Lind Alliance Priority Setting Partnerships | CHF | The Somerville Foundation | British Congenital Cardiac Association | SCTS Society for Cardiothoracic Surgery in Great Britain and Ireland

Have YOUR say on the future of Congenital Heart Disease research!

We are conducting a national study to identify and prioritise areas of future research in congenital heart disease that are most important to patients, their families, and healthcare professionals. With your help, the findings will have a major impact on the direction of congenital heart disease research in the UK— **so we want to hear from you!**

This project is focused on the how we treat and look after those with congenital heart disease throughout life, including prior to birth. We are specifically interested in your questions relating to:

- **Diagnosis**, during pregnancy or after birth
- **Treatment**, such as medical therapy (eg. drugs), catheter procedures, surgery, transplant, lifestyle and psychological or social help
- **Outcomes** of the conditions and/or treatments, and the impact on patients and their families, including the physical, psychological and social effects of living with congenital heart disease

We are not looking at areas such as causes of congenital heart defects, acquired heart disease in those without a congenital heart defect, or aspects of congenital disorders that do not affect the heart, which are outside of those above.

What questions would you like to see answered by future research, relating to the diagnosis, treatment, or outcomes of congenital heart disease? *Please enter up to three questions, one in each of the boxes below.*

Question 1:

Question 2:

Question 3:

Now we would like to know a bit about who you are and your interest in congenital heart disease, or the person on whose behalf you are completing this survey. *Please tick one box for each question below.*

What is your age (years)? ☐ 0-10 ☐ 11-15 ☐ 16-20 ☐ 21-35 ☐ 36-50 ☐ 51-65 ☐ 65+ ☐ Prefer not to say

What is your gender? ☐ Male ☐ Female ☐ Other ☐ Prefer not to say

What is your ethnicity? ☐ Arab ☐ Asian - Bangladeshi ☐ Asian - Indian ☐ Asian - Pakistani ☐ Chinese ☐ Any other Asian background ☐ Black - Caribbean ☐ Black - African ☐ Any other Black background ☐ White British ☐ White Irish ☐ Any other White background ☐ Mixed - White/Asian ☐ Mixed - White/Black ☐ Any other Mixed background ☐ Any other ethnic group ☐ Prefer not to say

Which of these best describes you? ☐ I was born with a congenital heart defect ☐ Parent ☐ Other relative ☐ Healthcare professional ☐ Charity ☐ Other - please specify:

Thank you for completing this initial survey. If given to you in clinic, please hand it in, or scan/photo and email to: congenitalPSP@contacts.bham.ac.uk, or post in an envelope to: **Freepost CONGENITAL PSP** (no stamp required). For more information, including on how we handle your personal data, please visit: www.birmingham.ac.uk/congenital-psp
