# Supplementary material for: Research priorities in children and adults with congenital heart disease: a James Lind Alliance Priority Setting Partnership
Source: Open Heart. 2022 Nov 22;9(2):e002147. doi: 10.1136/openhrt-2022-002147 (PMC9843188; doi:10.1136/openhrt-2022-002147)
Supplement: Supplementary data [file openhrt-2022-002147supp003.pdf]

Priorities for Congenital Heart Disease Research

Child/antenatal

Pre- and post-natal screening strategies

Minimise organ damage during surgery

Effects on brain development and behaviour

Long-term outcomes and life expectancy

Child/antenatal and Adult

Less invasive interventions

Longevity of the Fontan circulation

Impact on mental health

Technology to deliver personalised care

Need for repeated operations and interventions

Impact on quality of life

Adult

Pregnancy, childbirth and motherhood

Heart failure treatment

Management of arrythmias including sudden death

Transplantation and long-term mechanical support

James Lind Alliance Priority Setting Partnerships

CHF

Somerville Heart Foundation

British Congenital Cardiac Association

SCTS Society for Cardiothoracic Surgery in Great Britain and Ireland
